# Progress in Ophthalmology

**Published:** 1902-08-09

**Authors:** 


### Medical Progress.

### Progress in Ophthalmology.

**Some Functional Disorders of Vision.**—Mr. Henry Eales, of Birmingham, in the Middlemore Lecture,[4] gives a fine account of some of these disorders. He points out that for the recognition of these troubles we are at the mercy of the patient's account of the symptoms, as they have generally passed off before the doctor comes on the scene; and he says that patients are liable to be very inaccurate in their accounts—which is, we suppose, only natural, seeing the amount of worry they have at the time. He begins with scintillating scotoma—also known as teichopsia, on account of the zigzag lines often seen round the scintillating spots, like the walls of the Paris fortifications; also described under the title of "ophthalmic migraine," on account of its association with migraine. This affection, he points out, is very common indeed, much more so than is generally supposed, firstly, because many patients have become so accustomed to these recurrent attacks that rapidly pass off leaving no ill effects that they make no complaint of them; and secondly, because if they do complain, it is rather on account of the headache which usually follows this peculiar temporary visual disorder, which itself is not described to the medical attendant, while only the worst cases, probably, come to the notice of the ophthalmic surgeon; yet, even in his experience, this condition is by no means a rare one, such patients, on account of the pronounced eye symptoms, seeking his advice in hopes that their trouble is really due to some ocular disorder—for which we may be able to give them relief—a vain hope usually destined to end in disappointment. A graphic description of an attack is given as follows:—The attack begins by a sparkling spot or scintillation, which suddenly appears near the centre or fixation point of the field of vision of each eye, which is still present if the eyes are closed, this rapidly enlarges till he can hardly see at all, the scintillating central area is encircled by a bright zigzag line, and he is then only able to see very imperfectly large objects, the loss of vision is often accompanied by a feeling of giddiness. For a variable time, from a few minutes to half an hour this condition persists, when it rapidly passes off, the sight commencing to return at the part first affected; on the recovery of vision, headache sets in, gets worse and goes on till he is quite prostrated, indeed the visual attack is only the early symptom of an attack of migraine, which commonly but not invariably follows. He lays great stress on the length of time of these phenomena and says, I do not think I have ever known a patient to state that these visual phenomena have lasted more than three quarters of an hour in the most extreme cases, while ten minutes or a quarter of an hour is the time usually given, and in no case has he known the headache to precede the visual troubles. It is interesting and important to notice that, in a large proportion of these cases, the scintillating scotoma passes rapidly into a condition of homonymous hemianopsia—that is to say, the corresponding half of the field of vision in each eye becomes a blank. These attacks of hemianopsia last for ten minutes or a quarter of an hour usually and then rapidly pass off. On the hemianopsia passing off headache usually supervenes, passing on to an attack of migraine. In an early stage of a fainting fit, before loss of consciousness takes place, or in slight attacks not passing on to a loss of consciousness, it is not uncommon for patients to complain of this condition of homonomous hemianopsia. As to the cause of the attacks and the place affected, he thinks we must look to the central nervous system as the seat of the disturbance, and the nature of this disturbance is probably a localised anæmia of the visual centres, from vascular spasm, leading to a loss of function. From a study of the arrangement of the whole visual apparatus from the eyes to the visual centres, it will be obvious that homonymous hemianopsia can only be produced by some cause affecting the central nervous apparatus posterior to the chiasma. The evanescent and recurrent nature of these attacks point to a circulatory disturbance as the cause, while the loss of function, and the occurrence of these attacks during fainting points to anæmia of the nerve centres as the actual condition it sets up, the anæmia being due to vaso-motor spasm, *per se*, in the case of migraine, while in fainting the spasm is no doubt due to the invariable tendency of arteries to contract if the intravascular tension is lowered. The headache that accompanies these attacks points to disturbance in the cerebral vascular system. Such disturbance in the visual cortical centre of the occipital lobe, setting up an irritation in the visual elements of that centre which, in accordance with the laws of physiology, is referred by the patient to corresponding points in the visual field. As to the treatment of such attacks Mr. Eales says it resolves itself into the treatment of migraine for the most part, often a very unsatisfactory matter; he thinks, however, it is important to ascertain the exciting causes of attack and avoid them if possible. A timely attention to the premonitory symptoms of extreme hunger, or constipation, by giving a purgative will often ward off or mitigate an attack; and for this purpose saline aperients, especially one containing a large percentage of sulphate of soda, seems the best. He is fond of prescribing Carlsbad salts with some sulphate of soda added. Where over exertion, excitement, or fatigue seem to predispose to an attack, these should be avoided. Where sightseeing, such as going to picture galleries, causes attacks, this should be altogether avoided or only indulged in for a very short period at a time, it being better to pay two short visits than one long one. If there is some intolerance of light, as is often the case with these patients, smoked glasses should be worn whenever the least necessity for them arises. If any astigmatism or other optical defect or any want of muscular balance is present, endeavours should be made to correct these with suitable glasses, as in many cases excited by ocular strain, a great reduction in the frequency of attacks takes place, if the defect is corrected by means of glasses or prisms.

[4] Birm. Med. Rec., January 1902.

